# Camera Traps on Wildlife Crossing Structures as a Tool in Gray Wolf (*Canis lupus*) Management - Five-Years Monitoring of Wolf Abundance Trends in Croatia

**DOI:** 10.1371/journal.pone.0156748

**Published:** 2016-06-21

**Authors:** Lidija Šver, Ana Bielen, Josip Križan, Goran Gužvica

**Affiliations:** 1 Department of Biochemical Engineering, Faculty of Food Technology and Biotechnology, University of Zagreb, Zagreb, Croatia; 2 Gekom Ltd., Geophysical and ecological modeling, Zagreb, Croatia; 3 Department for Biomonitoring and Nature Protection, Oikon Ltd., Institute for Applied Ecology, Zagreb, Croatia; Sichuan University, CHINA

## Abstract

The conservation of gray wolf (*Canis lupus*) and its coexistence with humans presents a challenge and requires continuous monitoring and management efforts. One of the non-invasive methods that produces high-quality wolf monitoring datasets is camera trapping. We present a novel monitoring approach where camera traps are positioned on wildlife crossing structures that channel the animals, thereby increasing trapping success and increasing the cost-efficiency of the method. In this way we have followed abundance trends of five wolf packs whose home ranges are intersected by a motorway which spans throughout the wolf distribution range in Croatia. During the five-year monitoring of six green bridges we have recorded 28 250 camera-events, 132 with wolves. Four viaducts were monitored for two years, recording 4914 camera-events, 185 with wolves. We have detected a negative abundance trend of the monitored Croatian wolf packs since 2011, especially severe in the northern part of the study area. Further, we have pinpointed the legal cull as probable major negative influence on the wolf pack abundance trends (linear regression, r^2^ > 0.75, *P* < 0.05). Using the same approach we did not find evidence for a negative impact of wolves on the prey populations, both wild ungulates and livestock. We encourage strict protection of wolf in Croatia until there is more data proving population stability. In conclusion, quantitative methods, such as the one presented here, should be used as much as possible when assessing wolf abundance trends.

## Introduction

Gray wolf *Canis lupus* is one of the few large European carnivores. Until recently, its populations were in decline due to traditional and ongoing conflict with humans. However, modern conservation efforts have enabled people and predators to coexist in human-dominated landscapes, largely outside protected areas. The wolf is the second most abundant large carnivore in Europe distributed in 10 populations with estimated total number greater than 10 000 individuals. Despite the present prevailing positive trends, threats to wolf populations such as habitat loss and low public acceptance and persecution are still present [1–3].

Wolf packs that inhabit Croatia are a part of a Dinaric-Balkan population counting up to 5000 individuals. It extends from Slovenia in the north, across Croatia and Bosnia and Herzegovina, ending in the south of Dinaric mountains in Greece [1,2,4]. Although wolves used to inhabit the entire Croatian territory, numerous human activities (e.g. legal killings and reduced habitat quality and quantity) caused a rapid decline in their numbers which resulted in only about 50 individuals left [5,6]. In 1995, such an alarming situation prompted the authorities to protect the wolf in Croatia, as well as to develop the Wolf Management Plans [6,7]. Wolf numbers and distribution in Croatia are estimated annually, starting from 2005. Between the years 2006 and 2010 wolf population seemed stable—estimated number was slightly higher than 200 individuals inhabiting the mountain areas of Gorski Kotar and Lika, and also a part of Dalmatia, southern Croatia [8]. The main problem of wolf management in this period was poaching and a negative attitude of livestock breeders and hunters toward wolves. Therefore, a legal cull of up to 10% of the estimated population in the areas with highest negative impact on livestock was allowed. In this way, 113 animals were permitted to be culled between 2005 and 2012, out of which 77 were realized [9]. However, from the year 2010 onwards wolf numbers in Croatia dropped below 200 individuals and quota was abolished in 2013. Nevertheless, the negative trend continued, and in 2015 the lowest estimated abundance since 2005 was reported—156 wolves [8].

One of the prerequisites for long-term wolf conservation is continuous monitoring and census [1,2]. However, wolves are elusive animals that occupy large territories and live at low densities. This makes them an extremely challenging monitoring subject and a number of methods such as genetic monitoring, collecting wolf presence signs, telemetry, camera trapping, harvest data, damage statistics, interviews with local people, expert assessments, and summing of rough estimates in hunting grounds, are necessarily used. However, methods are not sufficiently coordinated between countries. An additional problem is that wolf monitoring is expensive and logistically demanding [1–3,8,10–13]. It is therefore hard to follow wolf trends on a population level, spanning different countries, and establishment of a standardized transboundary set of techniques is of critical importance [1].

One of the exact non-invasive methods used in the monitoring of wolves and other large carnivores is camera trapping [11,14–16], with a major drawback that employing cameras over large areas is very expensive [17]. Camera traps are often installed on wildlife crossing structures in order to monitor their usage by animals [18]. Since motorways are fenced and present barriers to animal movement, crossing structures funnel the animals. This increases the number of camera records over what would be expected with a randomly placed camera in an open area. Therefore, camera traps placed on wildlife crossing structures should be much more cost-effective for monitoring of rare and elusive animals.

**Fig 1:**
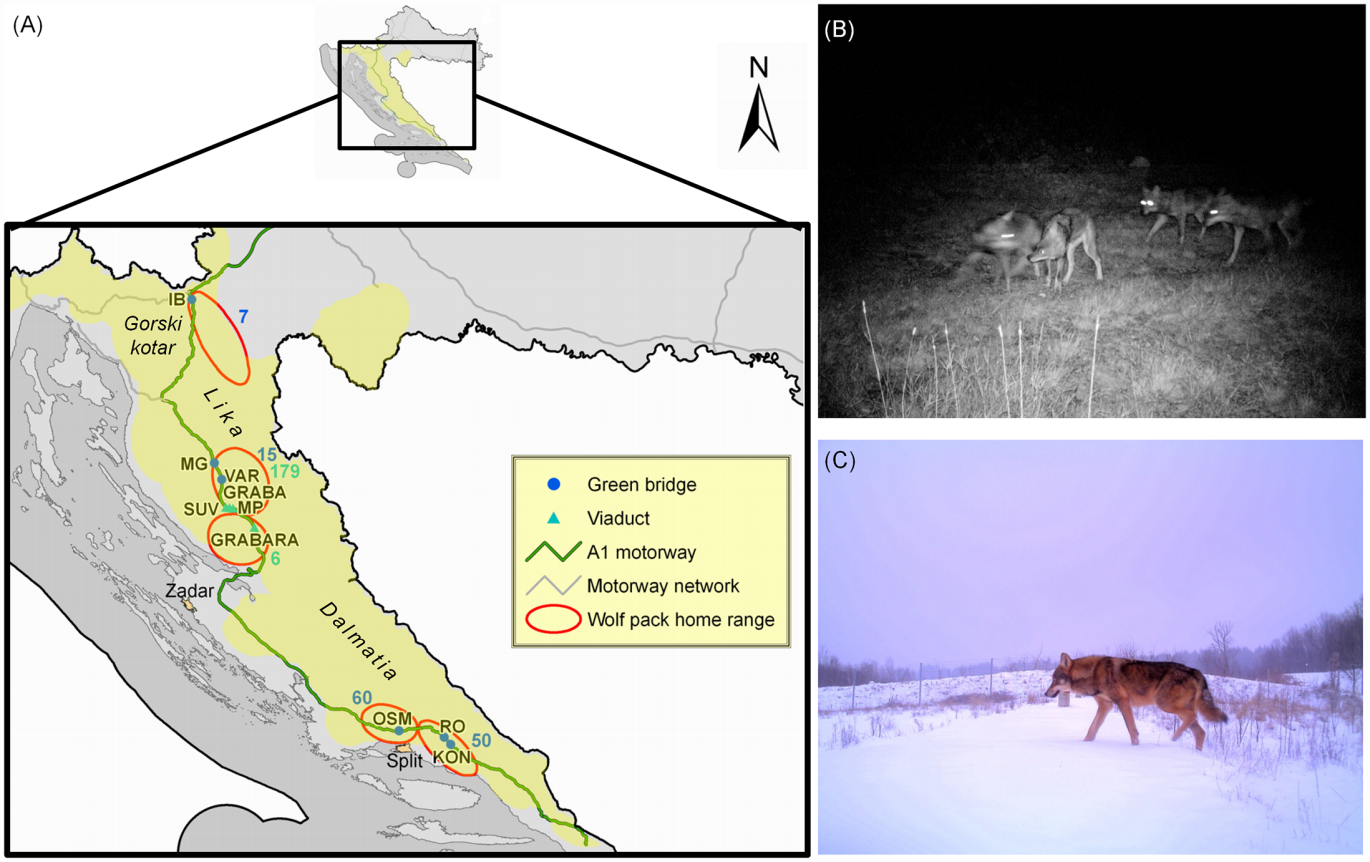
Investigated green bridges and viaducts. (A) Map of Croatia showing positions of the monitored green bridges and viaducts along the A1 motorway and wolf pack home ranges. Wolf range in Croatia is depicted in yellow. Total number of recorded photographs/movies with wolves on green bridges and viaducts is shown in blue and green, respectively. Map of Croatia representing motorway network was obtained from State Geodetic Administration (Republic of Croatia) and is used with their permission; (B) Night-time wolf pack photograph on the viaduct MP; (C) Daytime wolf photograph on the green bridge IB. Green bridges: IB—Ivačeno brdo, MG—Medina gora, VAR—Varošina, OSM—Osmakovac, RO—Rošca, KON—Konšćica; viaducts: GRABA—Graba, SUV—Suvaja, MP—Mandarića potok, GRABARA—Grabara.

Starting from this premise, we have employed camera trap data collected on wildlife crossing structures to estimate wolf abundance trends, assuming that the frequency of wolf crossings over the motorway is positively correlated with their abundance. In Croatia, crossing of the major A1 motorway (Fig 1) by various species has been monitored for years. The motorway passes through the wolf habitat and we have previously shown that the wildlife crossing structures on A1 are used by wolves [18]. Main objectives of this study were (i) to test the cost-efficiency of camera traps on wildlife crossing structures in monitoring wolf population trends, and (ii) to evaluate the role of legal harvest in the decreasing wolf abundance trends. Thus, we have followed abundance trends of several Croatian wolf packs during a five year period using the camera trap monitoring data from six green bridges and four viaducts. We demonstrate the cost-efficiency of such approach and we have identified the cull quota as a probable major negative influence on wolf packs in southern Croatia.

## Materials and Methods

### Study area and monitored wolf packs

The A1 motorway is a major north-south transportation corridor in Croatia (478.9 km, Fig 1A). Both sides of the motorway are fenced with approximately 2 m high wire mesh along entire length. Numerous wildlife crossing structures channel the animals, enable their migration across the motorway and minimize habitat fragmentation [18].

Our study included 325 km of A1 passing through the wolf range (Fig 1) [9,19]. Wild animal crossings were monitored for five years on six green bridges (Ivačeno brdo—IB, Medina gora—MG, Varošina—VAR, Osmakovac—OSM, Rošca—RO, Konšćica—KON) and for two years on four viaducts (Graba—GRABA, Suvaja—SUV, Mandarića potok—MP, Grabara—GRABARA) along the A1 (S1 Table). Wildlife crossing structures are protected by law as natural values (Nature Protection Act, OG 5/07). Permission to perform this research was obtained from the Ministry of Environmental and Nature Protection of the Republic of Croatia.

Lika and Gorski Kotar (Fig 1) are in the mountain region and continental climate zone, mostly covered with forests of beech *Fagus sylvatica* and fir *Abies alba*. This area is characterized by an abundance of wild ungulates, i.e. wild boar *Sus scrofa*, red deer *Cervus elaphus* and roe deer *Capreolus capreolus*. Dalmatia region is characterized by Mediterranean climate and thermophilic maquis of Mediterranean oak *Quercus ilex* and hornbeam *Carpinus orientalis*. Here, wolves prey mostly on livestock, since wild ungulates are present in much smaller numbers (mostly wild boar). Lika, Gorski kotar and inland Dalmatia are characterized by low human density which makes them suitable wolf habitats [6,20,21].

Wolves were recorded on all monitored structures. According to the literature [9,22–26], during the course of this study wildlife crossing structures were situated within the home range of five wolf packs: (i) wolf pack Saborsko was using IB; (ii) Golo trlo was using MG, VAR, GRABA, SUV and MP; (iii) Južni Velebit was using GRABARA; (iv) Vučevica was using OSM; and (v) Mosor was using RO and KON (Fig 1). Wolf packs are named throughout this manuscript according to the crossing structures they were using (Saborsko—IB, Golo trlo—MG+VAR / GRABA+SUV+MP, Južni Velebit—GRABARA; Vučevica—OSM, Mosor—RO+KON). Packs using the viaducts and green bridges IB, MG and VAR were situated in the mountain part of Croatia (*northern packs*), while the packs OSM and RO+KON were positioned southwardly in the Mediterranean climate zone (*southern packs*). Total home range of the five monitored wolf packs was calculated from the literature data [9,22–26] and amounted to 2453.4 km^2^. Therefore, this was the total monitored area in our study.

### Camera trap data collection and analysis

Utilization of crossing structures by gray wolf and its prey species (wild boar, red deer and roe deer) was monitored by digital camera traps. Four cameras with a passive infrared sensor (PIR) and IR light-emitting-diode flash (NoFlash, Cuddeback, Green Bay, WI, USA) were installed per green bridge, while the number of cameras positioned under viaducts varied according to their width (S1 Table). In total, 34 camera traps were used and checked approximately once a month. Cameras were in BearSafe (Cuddeback, USA) heavy duty metal cages, positioned at height of 0.5 m above ground and perpendicular to direction of animal movement. They operated continuously with minimal possible delay between two recordings (1 minute). Further details on camera settings are described in [18].

Monitoring year was defined as a period from April 1^st^ (beginning of the hunting/breeding season) to March 31^st^. Green bridges were being monitored for five years (app. 7300 trap days per bridge), and viaducts for two years app. 1460 trap days for GRABA, 2190 for SUV, 730 for MP, and 2920 for GRABARA; S1 Table). Rarely, monitoring had to be interrupted (maximum 3 months at a time) due to poor weather conditions that prevented normal functioning of cameras (e.g. high snow, low temperatures).

After a movement within the detection zone triggered the camera, it recorded one photograph and 30 second-movie. Presence and number of wolves and their prey were extracted from all photographs and movies. We used two parameters to analyze abundance trends of animals: (i) *event—*a photograph/movie with at least one wolf, wild boar, red deer or roe deer (regardless of the number of individuals observed in a single photograph/movie); and (ii) *minimal pack size*—maximal number of individuals observed in the single event for one year period. Occasionally, animals stayed for more than one min on the crossing structure, repeatedly triggering the camera. In order to minimize such redundancy, multiple events in the time frame of 10 min containing the same species and the same number of individuals were considered as a single event.

### External data analysis

We have used the available literature [22–26] to extract the data on wolf mortality and domestic animals killed by wolves for the counties that correspond to home ranges of monitored wolf packs (Karlovac County—IB, Lika-Senj County—MG+VAR, Split-Dalmatia County—OSM and RO+KON). We could clearly not achieve a perfect overlap between pack home ranges and selected counties. Nevertheless, this dataset complemented our camera trap data and put them in a wider context. We have calculated the percentage of domestic animals killed by wolves relative to the total number of registered livestock per year for the selected Counties. The data on wolf mortality was analyzed either as total wolf mortality per year for the selected County, or was further divided into accidental wolf casualties (including collisions with vehicles, poaching, poisoning and unknown causes) and legal cull.

### Statistical analysis

We have used simple linear regression to evaluate the impact of monitored wolf packs on prey populations, as well as the impact of anthropogenic activities on wolves [27] (*P* ≤ 0.05). We have pooled the data for ecologically homogeneous northern packs (IB and MG+VAR) and analyzed them separately from the data for southern packs (OSM and RO+KON). We tested the assumption that the number of events and minimal pack size are positively correlated, assuming that a higher number of events should reflect a larger pack that occupies a particular area. This was true for southern packs (r^2^ = 0.74, *P* < 0.05), while no statistically significant correlation could be found for the northern packs (r^2^ = -0.16, *P* = 0.55). Therefore, we concluded that the number of events with wolves (22 out of 15 893) and estimated minimal pack sizes (1–3) were in the northern part of the study area too small for reliable statistical analysis, and regression analyses were only made for more abundant southern packs (i.e. higher observed number of events with wolves, 217 out of 10 699, and estimated minimal pack sizes between one and eight).

## Results

### Abundance trends of monitored wolf packs

**Fig 2:**
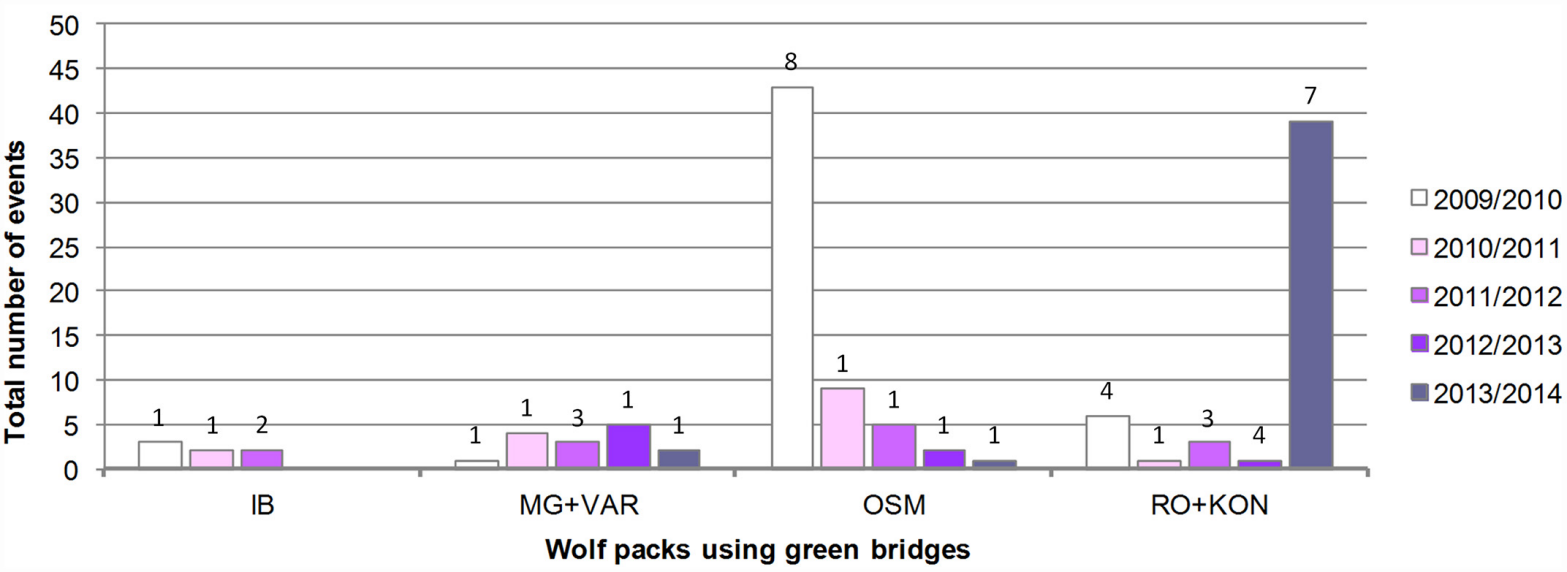
Abundance trends of wolf packs using monitored green bridges. Event—a photograph/movie of a wolf/wolves. Maximal number of wolves recorded in a single event for each year, i.e. minimal pack size, is marked above bars.

**Fig 3:**
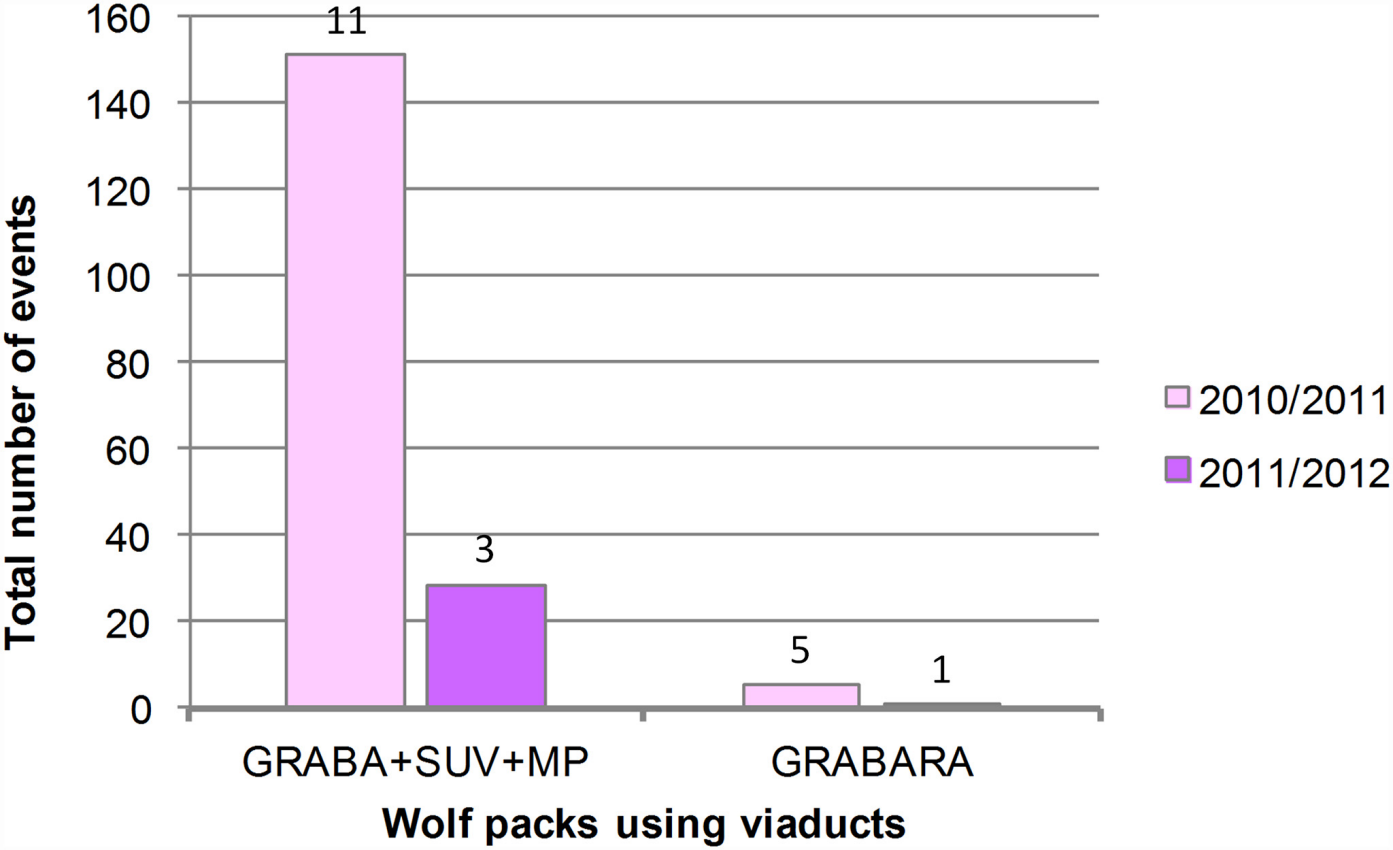
Abundance trends of wolf packs using monitored viaducts. Event—a photograph/movie of a wolf/wolves. Maximal number of wolves recorded in a single event for each year, i.e. minimal pack size, is marked above bars.

We have followed abundance trends of five Croatian wolf packs whose home ranges are intersected by A1 [22–26]. During monitoring period we have recorded and analyzed 28 250 camera-events on green bridges. 132 events (0.47%) contained wolves: IB—7 / 7721 (0.09%), MG+VAR—15 / 7960 (0.19%), OSM—60 / 6386 (0.94%), RO+KON—50 / 6183 (0.81%). Viaducts were monitored for two years, and we have recorded and analyzed 4914 camera-events. 185 events (3.76%) contained wolves: GRABA+SUV+MP—179 / 3930 (4.55%), GRABARA—6 / 984 (0.61%). Abundance trends of wolf packs that used the crossing structures during the monitoring period are presented in Figs 2 and 3. Northern packs IB, MG+VAR / GRABA+SUV+MP, and GRABARA mostly appeared in low numbers. Wolf pack IB was even completely absent from the April 2012 till the end of the monitoring. Further, decrease in number of events and minimal pack size was evident for both packs using viaducts. From 2011/2012 onwards a very small number of wolves were recorded in the whole northern monitoring area (green bridges IB, MG and VAR, and all four viaducts). The only exception was the season 2010/2011 when the minimal estimated size for the pack MG+VAR / GRABA+SUV+MP was 11 (recorded on MP). A similar negative trend, illustrated by a sharp drop in number of events and minimal pack size from 2010/2011 onwards, was seen on the Mediterranean green bridge OSM. The southernmost RO+KON pack was the only one that showed signs of recovery by the end of the monitoring period (2013/2014).

### Prey abundance trends in the habitat of monitored wolf packs

**Fig 4:**
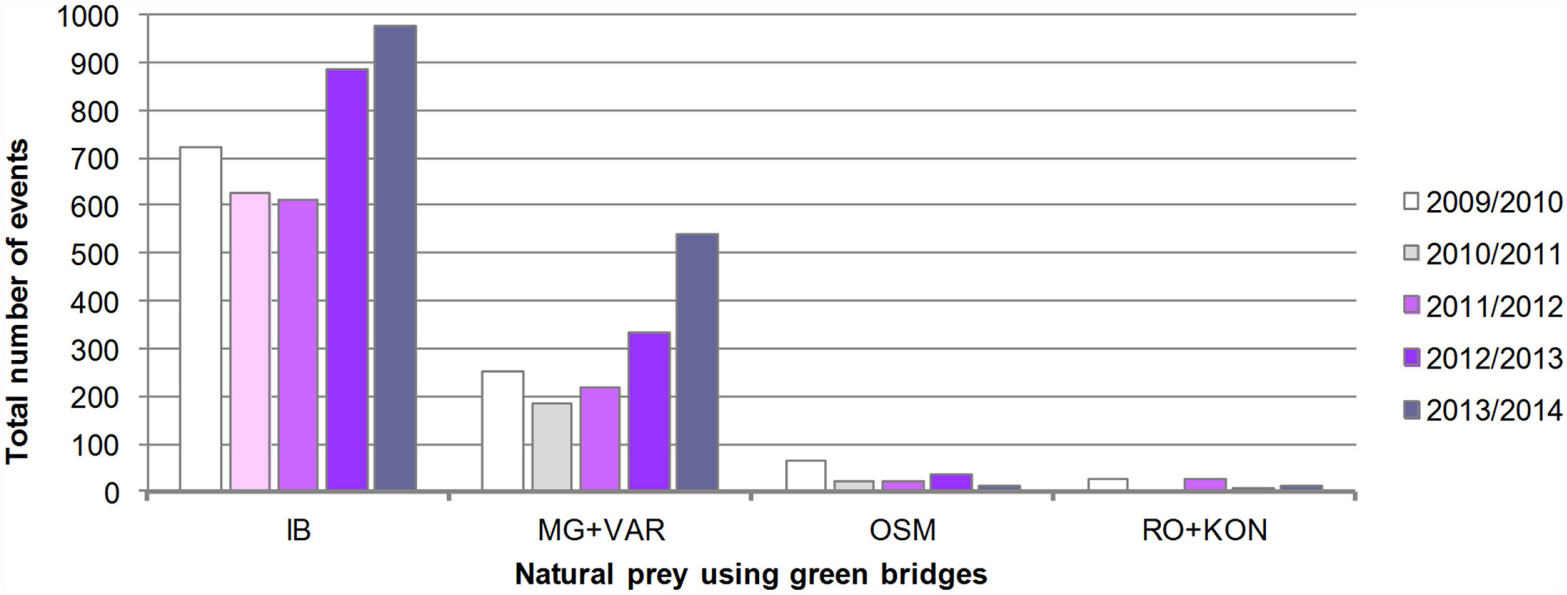
Abundance trends of natural prey (roe deer, red deer and wild boar combined) on the monitored green bridges. Event—a photograph/movie of natural prey.

We have analyzed abundance trends of wolf's natural prey in home ranges used by wolf packs IB, MG+VAR, OSM and RO+KON. We used the same dataset as was used for the analysis of wolves—28 250 camera-events collected on green bridges, and processed them to calculate the number of events and minimal herd size. In total, 5570 out of 28 250 camera-events (19.7%) contained wild boar, red deer or roe deer: IB—3824 / 7721 (49.5%), MG+VAR—1529 / 7960 (19.2%), OSM—156 / 6386 (2.4%), RO+KON—61 / 6183 (1.0%). Occurrence of natural prey species was much higher in the territory of two northern packs (IB and MG+VAR), compared to the territory of southern packs OSM and RO+KON (Fig 4). Furthermore, separate analysis for each prey species showed that the roe deer represents a dominant prey species in the IB and MG+VAR territory, while the wild boar and the red deer are present in much smaller numbers (S1 Fig). On the contrary, only the wild boar is present in significant numbers in the area inhabited by southern packs, while the red deer and the roe deer are completely or mostly absent. Further, in the territory of northern packs, a trend of increase in the number of natural prey events was easily observable in the last two years of monitoring (Fig 4), primarily due to the roe deer events (S1 Fig).

Wolves in Croatia often prey on domestic animals [20]. Since domestic animals mostly did not use the green bridges, we have complemented our analysis with external data. We have calculated the percentage of domestic animals killed by wolves relative to the total number of registered livestock for the selected Counties (S2 Fig). In Karlovac and Lika-Senj counties, corresponding to the territories of IB and MG+VAR packs, this percentage was below 0.25%. On the contrary, for Split-Dalmatia County inhabited by southern packs OSM and RO+KON, we have observed app. four times higher percentage of domestic animals killed by wolves (~ 1%).

### Absence of negative impact of monitored wolf packs on the available prey

We have examined whether wolf abundance trends correspond to changes in prey abundance for the more abundant southern packs. We did not find a statistically significant correlation between wolf and prey abundance trends, both for wild prey and livestock (*P* > 0.05; Table 1).

**Table 1:** Testing the correlation between abundance trends of wolves and (i) their prey and (ii) human-caused wolf mortality in the southern part of wolf range (packs OSM and RO+KON).

Dependent variable	Independent variable	Adjusted r^2^	t	*P*
**number of wolf events**	total number of natural prey events	0.07	1.15	0.33
	number of domestic animals killed by wolves^a^	0.17	1.36	0.27
**minimal wolf pack size**	total number of natural prey events	0.47	2.12	0.12
	number of domestic animals killed by wolves^a^	0.67	3.02	0.06
	total mortality^a^	-0.21	-0.55	0.62
**number of wolf events**	accidental casualties^a^	0.13	1.26	0.30
	legal cull^a^	0.87	-5.28*	0.01*
	total mortality^a^	-0.15	-0.70	0.54
**minimal wolf pack size**	accidental casualties^a^	-0.03	0.93	0.42
	legal cull^a^	0.77	-3.84*	0.03*

^a^Data for the corresponding Split-Dalmatia County and time period were extracted from the literature. Total wolf mortality = accidental wolf casualties + legal cull.

* *P* < 0.05.

### Negative impact of human-caused wolf mortality on the monitored wolf packs

**Fig 5:**
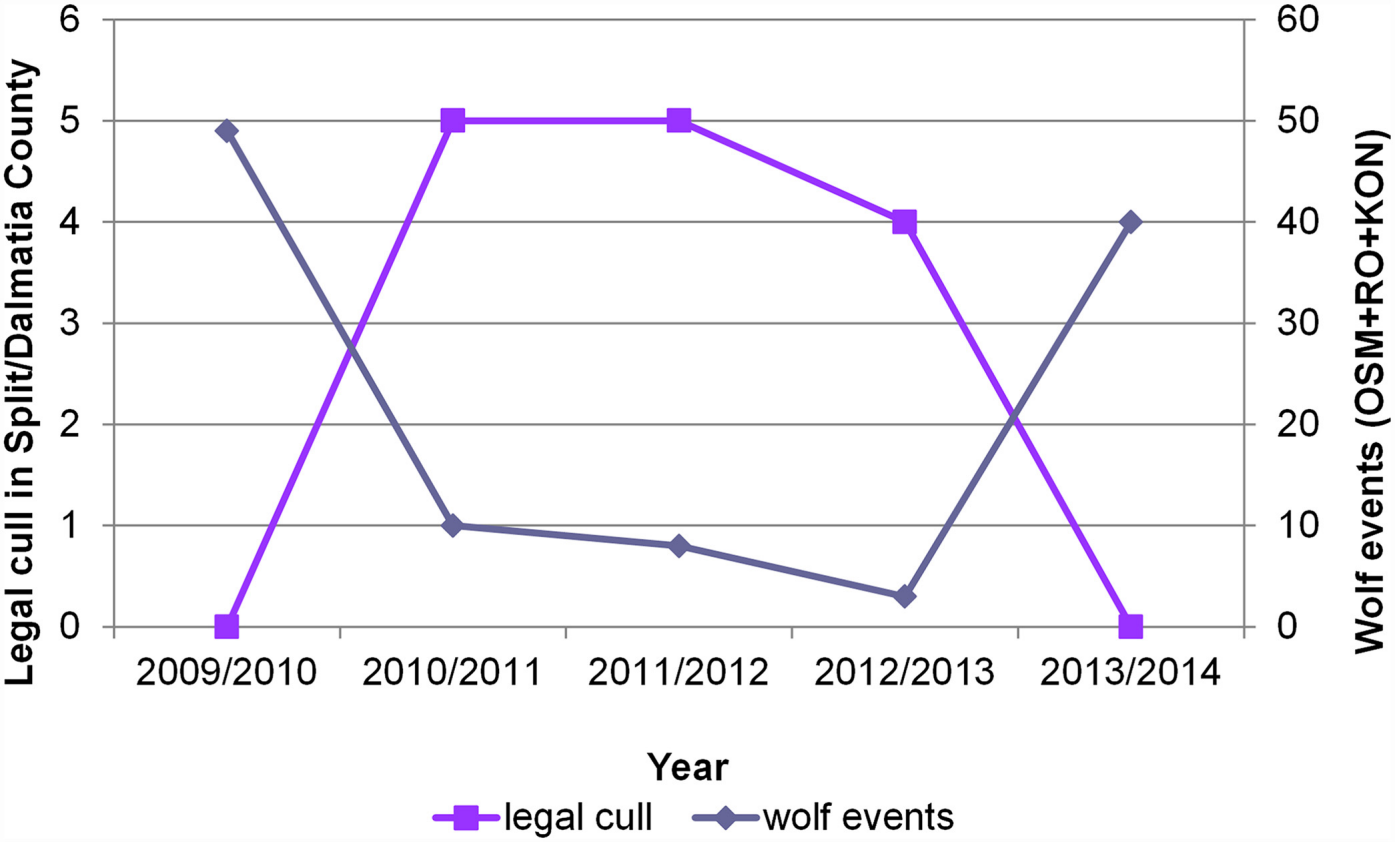
Negative correlation between wolf abundance trends and legal wolf cull in southern Croatia. Wolf population trend is presented as a total number of events (photographs of wolf/wolves) recorded on Dalmatian green bridges. The data for the legal cull in the corresponding Split-Dalmatia County during the monitoring period was extracted from the literature.

We have investigated possible correlations between wolf abundance trends and human-caused wolf mortality in the southern part of wolf range in Croatia (Table 1). Total wolf mortality was divided into legal cull (34,1% of total mortality) and accidental wolf casualties (65,9% of total mortality). Accidental wolf casualties included 70,4% collisions with vehicles, 22,2% poaching, 3,7% poisoning and 3,7% unknown causes. We did not find a statistically significant correlation between the number of wolf events/minimal pack size and accidental wolf casualties or total wolf casualties. However, a statistically significant and negative correlation was found between the number of wolf events/minimal pack size and the number of legal cull, where with linear regression we could explain more than 75% of the variation (r^2^ > 0.75, *P* < 0.05). A high number of legal cull corresponded with a low number of wolf events recorded on OSM, RO and KON (Fig 5).

## Discussion

### Efficiency of camera traps on green bridges and viaducts in wolf monitoring

We used camera trapping for the five-year monitoring of wolves in Croatia. Camera trapping allows precise species identification and has often been used in monitoring of large carnivores [1,11,14–16,28]. In order to increase the trapping efficiency, cameras are usually positioned in a raster arrangement and either equipped with a bait [11,15] or placed in an area known to be used by target individuals [14,16]. Contrary to this, we positioned the camera traps linearly, i.e. on wildlife crossing structures along the major motorway passing through the wolf range in Croatia. We used this camera trapping design to follow wolf abundance trends. To confirm the efficiency of the method, it was important to include a positive control—namely, external data on the number of wolves living in the area of at least one crossing structure. In our case, possible source of such data are annual estimates of wolf abundance per pack published by State Institute for Nature Protection (SINP, Republic of Croatia) [9,22–26]. However, these assessments are, apart from harvest data and damage statistics, largely based on non-quantitative methods [such as collecting wolf presence signs, interviews with local people (non-experts), expert assessments and estimates in hunting grounds]. Therefore, we have used another line of evidence to choose which SINP pack census should be reliable: an unpublished telemetry dataset of male wolf that we monitored for 14 months during 2009 and 2010 (at the beginning of the monitoring period presented here). This GPS-collared wolf was captured in the vicinity of green bridge Osmakovac and our unpublished data show it crossing the green bridge. Thus, we have compared the sizes of wolf pack OSM (Vučevica) estimated by SINP over the monitoring period with our data and show that these two curves fit well (S3 Fig). Further, our results show a general negative abundance trend of the monitored wolf packs since 2011 and this is in agreement with the annual estimates of wolf abundance for the whole Croatia between 2009 and 2014: 215 individuals in 2009, 230 in 2010, 193 in 2011, 198 in 2012, 177 in 2013, and 168 in 2014, distributed in app. 50 packs [8,9]. Therefore, the negative trend observed here for five wolf packs (app. 10% of the Croatian population) coincides with the overall decrease of the wolf number in Croatia, confirming the usefulness of our methodology.

The underlying rationale of our method is that changes in the frequency of camera events can be ascribed to the changes in animal abundance. This should be true if animals do not significantly change their behavior/usage of crossing structures throughout the monitoring period. Thus, target individuals have to be already habituated to the crossing structures at the beginning of the monitoring and their home ranges should stay more or less unchanged throughout the study period. Both requirements were fulfilled here, as explained below.

Habituation time varies depending on the species, and it can take up to several years for animals to adjust their behavior to crossing structures in their habitat. The adaptation period of wolves in Banff National Park (Canada) was approximately five years [29]. In our case, majority of monitored crossing structures were built five or six years before the start of the study, making it reasonable to assume that wolves were already adapted to their presence. The only exceptions were RO and KON that were built 2.5 years before the beginning of the study and therefore we cannot exclude the possibility that low number of recorded events in the first two to three monitoring years is due to still ongoing habituation.

Our approach should be well suited for wolf monitoring since wolf packs were shown to have stable home ranges for as much as 10 years [30–32]. Based on the available literature on wolf pack home ranges in Croatia and elsewhere, their size and the fact that they are not overlapping, we assumed that monitored green bridges were used throughout the five-year monitoring period by the same wolf packs [9,20,22–26,33]. Consequently, when we detected a significant decrease in number of crossings, we assumed that the cause was the decrease in pack size, and *vice versa*. Of course, it is possible that some wolves (e.g. dispersing individuals) migrated away from the crossing structure and therefore stopped appearing in recordings later on (due to altered behavior and not to death), but such events should be rare and should not significantly affect overall wolf pack abundance trends.

Noteworthy, our monitoring setting can be applied to follow abundance trends, but not to make a quantitative estimate of wolf density (e.g. to estimate wolf abundance). Firstly, recognition of individuals, crucial for capture-recapture statistical modeling of population sizes, was not possible due to the absence of distinctive individual markings in wolves. Camera traps are mostly used to estimate the abundance of felids with coat patterns enabling individual recognition [34]. Further, external conditions (such as night-time, heavy rain or fog) often negatively affected the quality of recorded photographs/movies, and recognition of details was not possible. Although the method for density estimation without the need for individual recognition exists [35], it could not be applied here because cameras were not placed randomly with respect to animal movement.

In conclusion, the method presented here has many advantages over the traditional camera monitoring setups, as summarized in Table 2. Most importantly, the long-term expenses of our setup are acceptable because the same dataset can be used in multiple monitoring studies. Namely, crossing structures act as funnels that channel diverse animals, without the need to aim at a particular target species. Cost reduction should be especially significant when monitoring scarce animals with large home ranges such as wolves. To show the cost efficiency of our method, we have compared our setup with camera trapping designs from the literature (Table 3). In general, cost of the study lowers (i) with the reduction in the number of camera traps per km^2^ and (ii) with increasing intervals between field camera trap data collection. Both parameters are highly cost-effective in our case, compared to other monitoring designs (Table 3)—we have used only 0.01 camera traps per km^2^ and frequency of field trips was only once a month. Despite these low costs, we were able to estimate abundance trends of five wolf packs.

**Table 2:** Benefits and limitations of camera traps on crossing structures used as a wildlife monitoring tool.

**Benefits**	Increased trapping probability in comparison to randomly placed cameras due to funnel effect	+++
	Cost reduction in comparison to randomly placed cameras due to funnel effect	+++
	Cost-efficient monitoring of large carnivores' abundance trends (i.e. wolves and other animals with large home ranges)	+++
	Simultaneous monitoring of highway permeability for diverse wildlife species	+++
	Possibility to determine minimal number of wolves in the pack	++
**Limitations**	Not applicable for newly built crossing structures (5 years of habituation recommended for carnivores)	- - -
	Crossing structures positioned at the home range boundaries could have lower crossing frequency	- -
	Fast moving animals can occasionally be missed (e.g. roe deer) [18]	-

Number of +/- indicate the strength of benefit/limitation.

**Table 3:** Cost-efficiency of various camera trapping studies.

Camera trapping design	Total number of camera traps	Monitored area (in km^2^)	Number of camera traps per km^2^	Frequency of camera visits (in days)	Aim of the study	Ref.
Camera traps located at wildlife crossing structures that act as funnels	34	2453.4*	0.01	30	Monitoring of wolves with estimation of abundance trends	This study
Camera traps located at crossroads or close to recent wolf fecal marks, relocated every 3–30 days	5	49.74	0.10	3–30	Monitoring of wolves	[14]
Raster arrangement of camera traps (regular grid with relocation every 30 days)	20–30	120.0–16183	0.001–0.25	30	Diversity of tropical forest mammals	[36]
Raster arrangement of camera traps, 1–1.5 km apart, on existing trails ("natural funnels")	26	26.9	0.97	14	Evaluating camera trapping success for different carnivores	[37]
Camera traps positioned in a grid, 1 km apart, at sites with animal signs	56	54	1.04	1	Camera-trapping to determine maned wolf density	[15]
Linear arrangement of camera traps along the road, 1.5 km apart	29	21.8	1.33	n. r.	Comparison of different methods to determine species richness and abundance	[38]
Camera traps distributed randomly and baited	4	2.5	1.60	3.5	Comparison of methods for monitoring of carnivores	[11]
Randomly spaced camera traps, relocated every 10 days	6	0.49	12.24	1	Estimating density of different species	[35]

n. r.—not reported

*total home range of five monitored wolf packs

### Evaluating the role of legal cull in the descending wolf abundance trends

Using described methodology we confirmed that northern and southern wolf packs lived in ecologically different situation, primarily because of differences in available prey. Most important wolf's natural prey in Gorski Kotar and Lika are wild ungulates [6,20,21]. We confirmed that prey of the northern packs are roe deer, wild boar and red deer. This is evident from the high number of events of natural prey on IB, MG and VAR and low percentage of domestic animals killed by wolves in Karlovac and Lika-Senj Counties. Further, the trend of an increase in the number of natural prey events (mostly roe deer) was observable in the last two monitoring years on IB, MG and VAR. This coincides with very small number of recorded wolves for these locations and period, and implies that small number of wolves could contribute to the increase in prey population size. The importance of wolf predation in limiting reindeer *Rangifer tarandus* population growth in Finland was reported previously [39]. However, in our case no statistically significant correlation between number of wolf events / minimal wolf pack size and total number of natural prey events could be established.

Low abundance of natural prey can stimulate wolves to prey on livestock [40]. Our data confirm that only limited number of wild boars is available as a natural prey in Dalmatia (OSM, RO and KON). Therefore, southern packs presumably preyed mostly on domestic animals, as supported by higher percentage of domestic animals killed by wolves in Split-Dalmatia County (app. 1%), compared to northern Karlovac and Lika-Senj counties (< 0.25%). Previous analysis of stomach content of dead wolves and of wolf feces in Dalmatia also showed that their diet consists predominantly of domestic animals [20]. Despite this, we did not find evidence for a negative impact of wolf packs OSM and RO+KON on the available wild prey and livestock (absence of negative correlation between wolf abundance trends and prey population trends, *P* > 0.05).

One of the most important obstacles in wolf management is traditionally low public acceptance, further provoked by wolf predation on livestock [6,7,41,42]. Addressing these public concerns is the reason why many countries allowed legal harvest of moderate percentage of population [6,7,41,43,44]. As already mentioned, legal harvest of 10 to 15% of estimated wolf abundance was allowed in Croatia from 2005 to 2012 [8]. However, we present a negative abundance trend of the monitored Croatian wolf packs since 2011. The wolf abundance parameters were especially small for northern packs. This was particularly alarming for the wolf packs Golo trlo (MG+VAR / GRABA+SUV+MP) and Južni Velebit (GRABARA), with home ranges located in the centre of wolf distribution in Croatia. The most drastic example was green bridge IB, where we did not detect wolves after 2011. Further, we have established a statistically significant and negative correlation between number of wolf events / minimal pack size and number of culling (*P* < 0.05), while no statistically significant correlation was found between the wolf abundance parameters and accidental wolf casualties. This indicates that the legal harvest is a probable major cause of the negative wolf abundance trend during the monitoring period, at least in Dalmatia. Importantly, legal cull constituted large part of total wolf mortality during our monitoring period (app. 1/3) and it is a part of wolf mortality that can be most easily removed. Therefore, our results support the decision of the authorities to abolish the cull quota in 2013 [8]. For the northern part of the study area, statistical analysis was hampered by an extremely low number of wolf events, but the negative anthropogenic influence was probably even more pronounced there. Our results are in agreement with the study of North American wolf populations by Creel and Rotella [44] showing a strong association between human offtake and total wolf mortality rate. There are several possible reasons why a legal harvest, predicted not to pose a threat to the population, nevertheless could cause a population decline, as was the case here. Firstly, the wolf abundance could have been overrated in official reports. Namely, human assessments often cause overestimation of wolf numbers, due to the multiple counting of the same packs in neighboring census units [1,31]. Consequently, the assessed cull quota could have also been too high. Quantitative methods (as the one presented here) should be used as often as possible when assessing population size, as opposed to subjective assessments prone to errors. Secondly, significant number of unrecorded illegal wolf killings has probably occurred [8], inflicting further damage. The additional point to be discussed is the period required for the recovery of Croatian wolf packs after cancelling the cull quota in 2013. From 2013 up to now, the negative abundance trend continued, visible both on the country level (156 wolves in 2015 in Croatia [8]) and from our study. Namely, for the northern packs there were no signs of immediate recovery, presumably because of very low population density at the moment of cancelling the cull quota. The only pack where we could detect an immediate positive effect of cancelling the cull quota was Dalmatian RO+KON pack. Wolves were again recorded in the second half of the 2014 in higher number on OSM too (minimal pack size—3; data not shown). Therefore, we can conclude that our method could detect the signs of recovery on the level of individual wolf packs, when this could still not be observed on the country level.

## Conclusions

We describe a unique approach in camera trap monitoring—linear arrangement of camera traps on wildlife crossing structures positioned over fenced motorways. In this way, wildlife crossing structures can serve as an excellent long-term wildlife monitoring stations for the species with large and stable home ranges over prolonged periods, such as wolves. Using this approach we did not find evidence for a negative impact of monitored Croatian wolf packs on the available prey. However, we have established a negative correlation between the cull killings and wolf abundance trends. We encourage other researchers and conservationists to use here developed cost-effective approach in the monitoring of wolves and other wildlife species.

## Supporting Information

S1 Fig Abundance trends of natural prey on the monitored green bridges. Event—a photograph/movie. Maximal number of animals recorded in a single event for each year, i.e. minimal herd size, is marked above bars.
(DOC)

S2 Fig Percentage of domestic animals (cattle, goat, sheep, horse and donkey) killed by wolves (relative to the total number of registered animals per county). Counties were selected according to the presumed territories of four wolf packs monitored in this study: Karlovac—IB, Lika-Senj—MG+VAR, Split-Dalmatia—OSM and RO+KON. Data were taken from the available literature [8,9,22–26].
(DOC)

S3 Fig Decrease of the wolf pack OSM during the monitoring period—comparison of estimates made in this study and official estimates (State Institute for Nature Protection). (DOC)

S1 Table Monitored wildlife crossing structures. (DOC)

S2 Table Data used in this study. (XLSX)
