# Supplementary figures and images for: Camera Traps on Wildlife Crossing Structures as a Tool in Gray Wolf (Canis lupus) Management - Five-Years Monitoring of Wolf Abundance Trends in Croatia
Source: PLoS One. 2016 Jun 21;11(6):e0156748. doi: 10.1371/journal.pone.0156748 (PMC4915698; doi:10.1371/journal.pone.0156748)

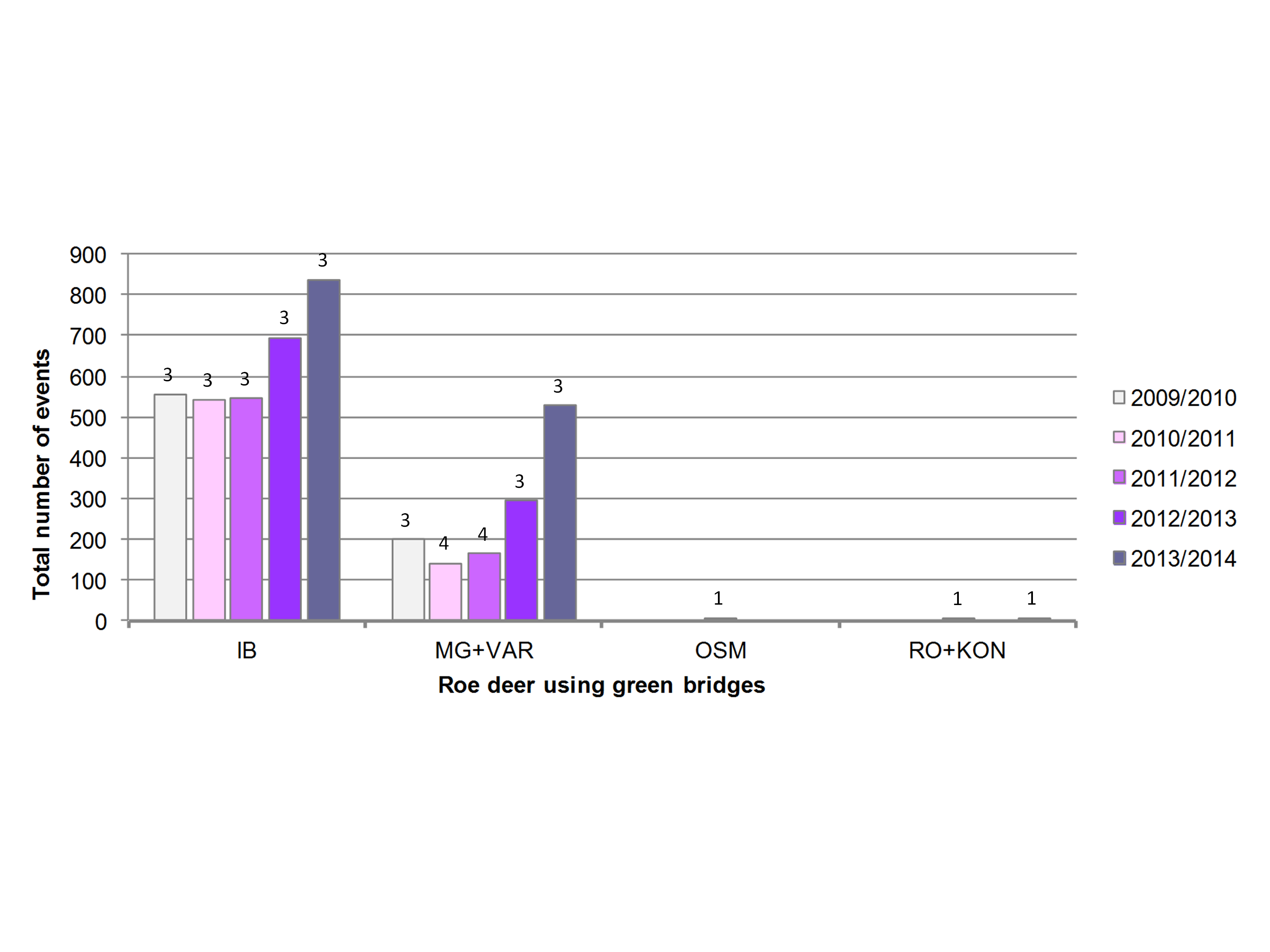

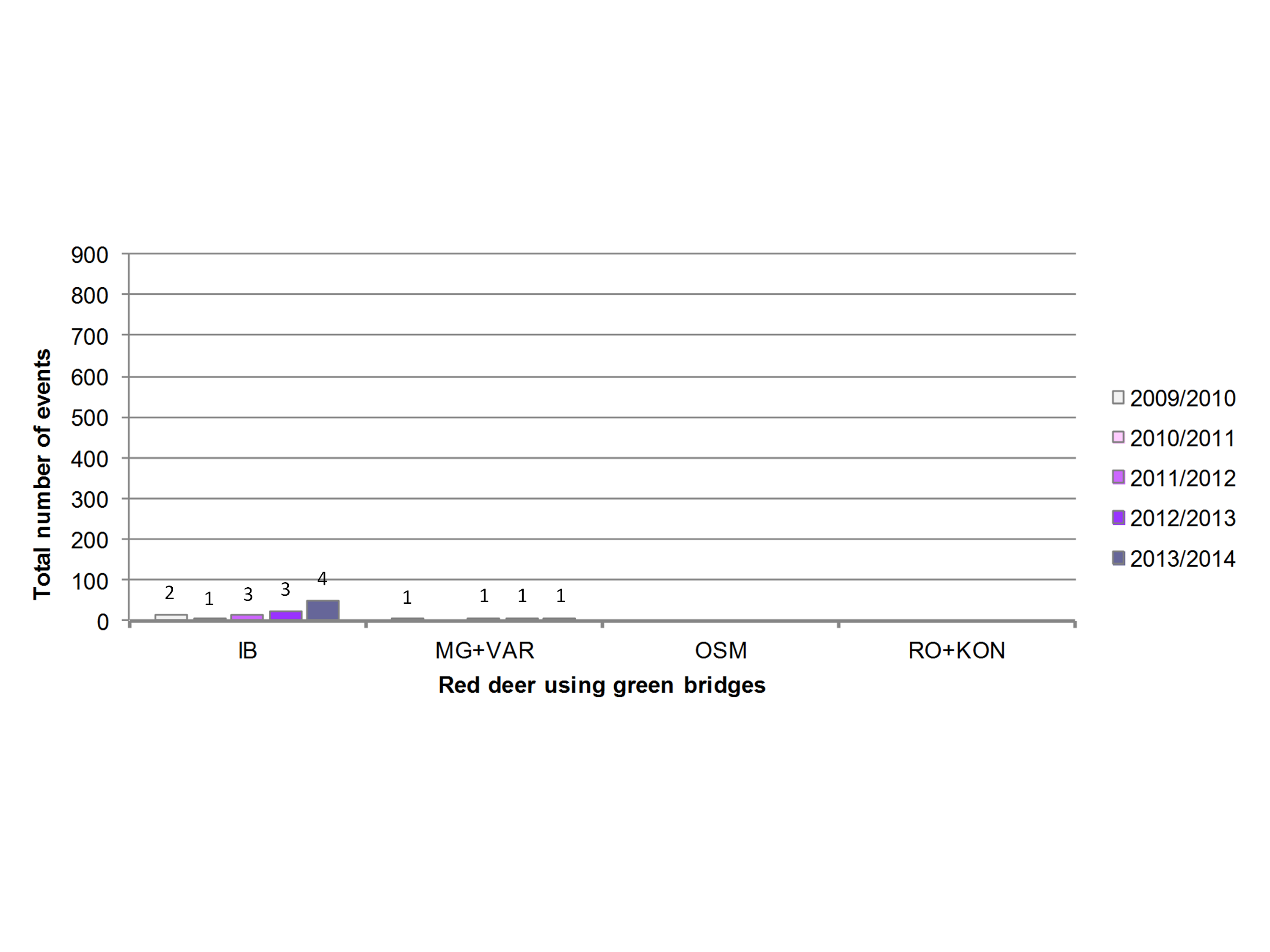

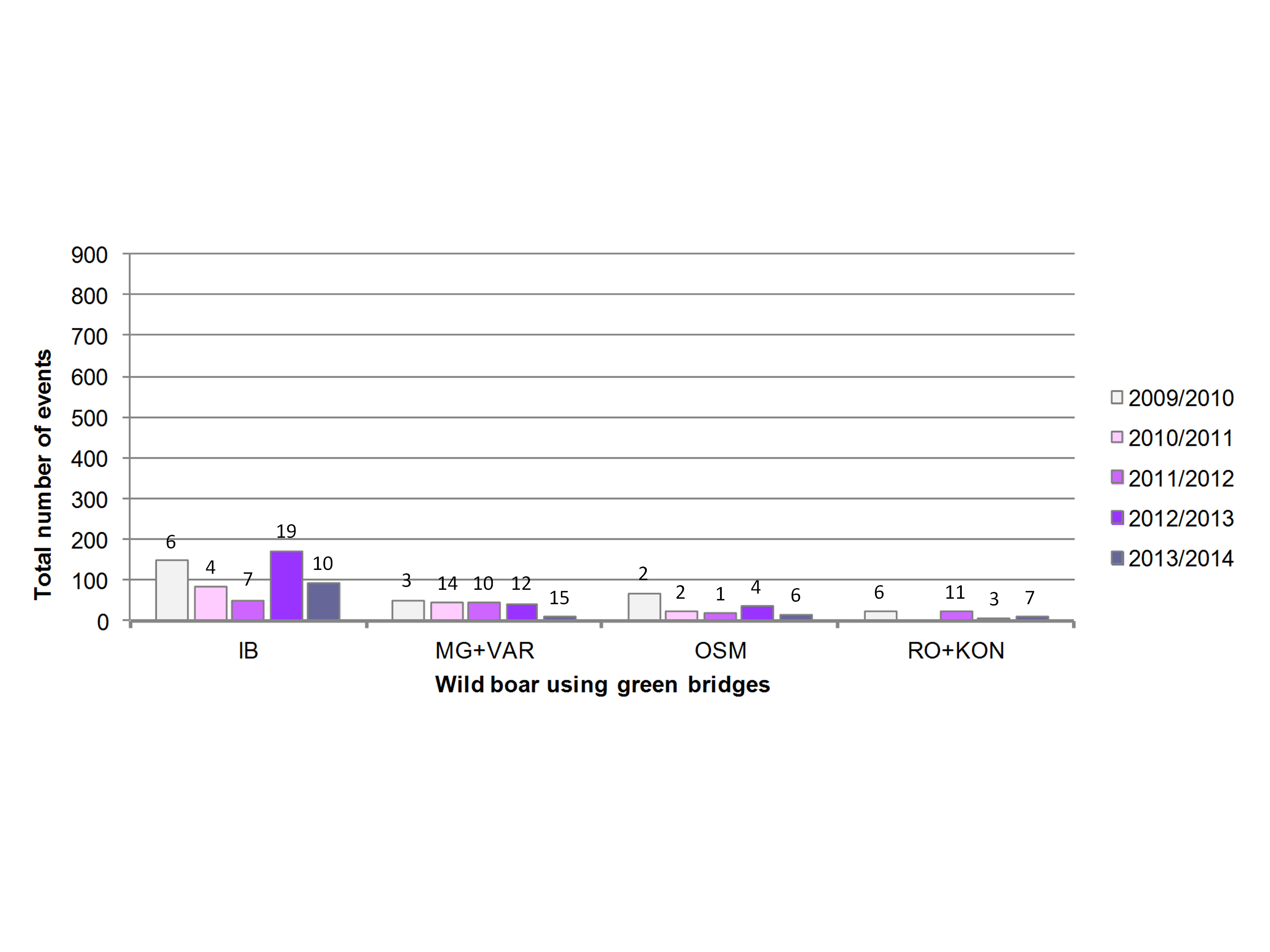


(b)

(a)

(c)

(c)

Supplement: S1 Fig — Event—a photograph/movie. Maximal number of animals recorded in a single event for each year, i.e. minimal herd size, is marked above bars. (DOC) [file pone.0156748.s001.doc]

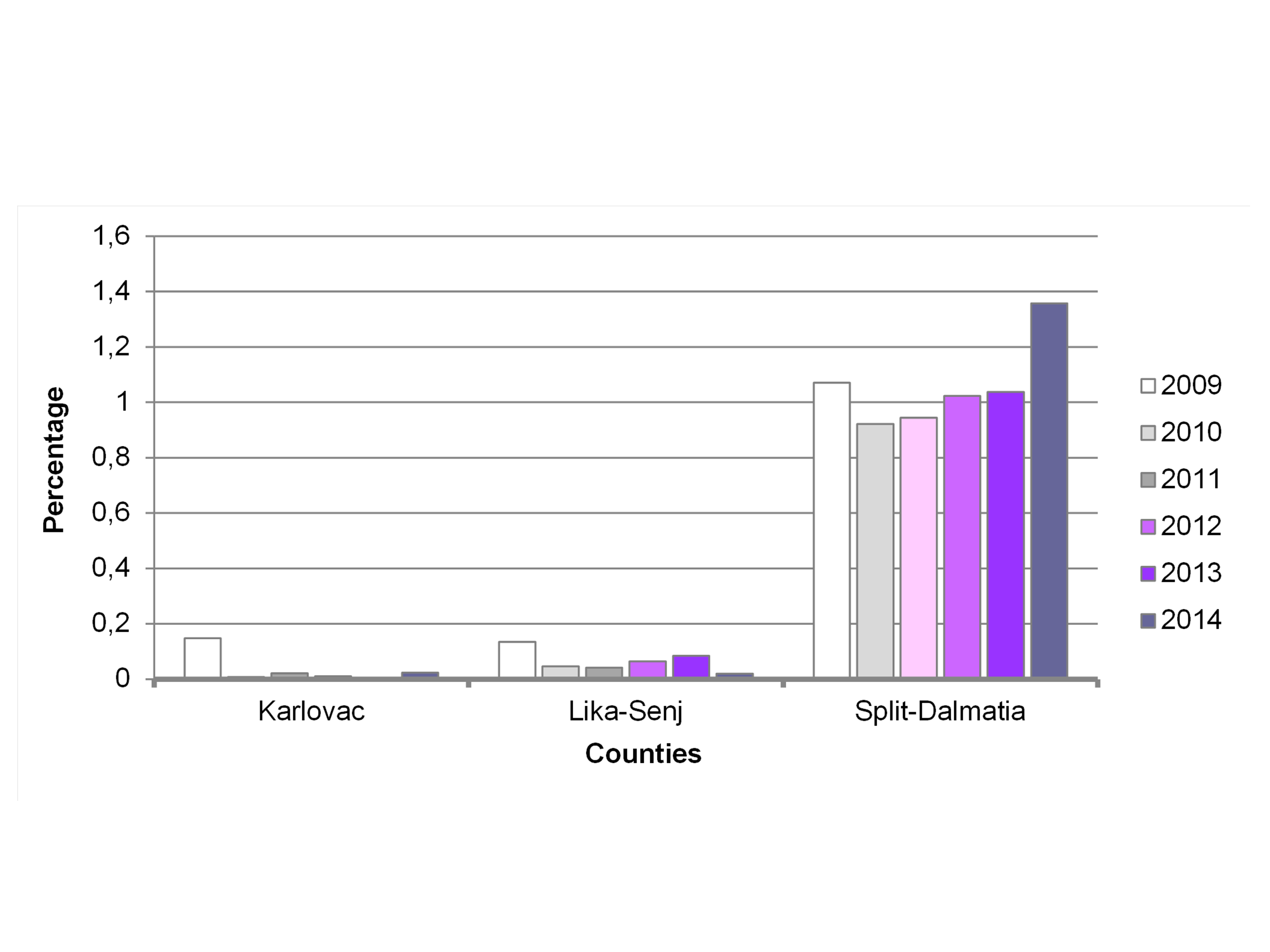

Supplement: S2 Fig — Counties were selected according to the presumed territories of four wolf packs monitored in this study: Karlovac—IB, Lika-Senj—MG+VAR, Split-Dalmatia—OSM and RO+KON. Data were taken from the available literature [8,9,22–26]. (DOC) [file pone.0156748.s002.doc]

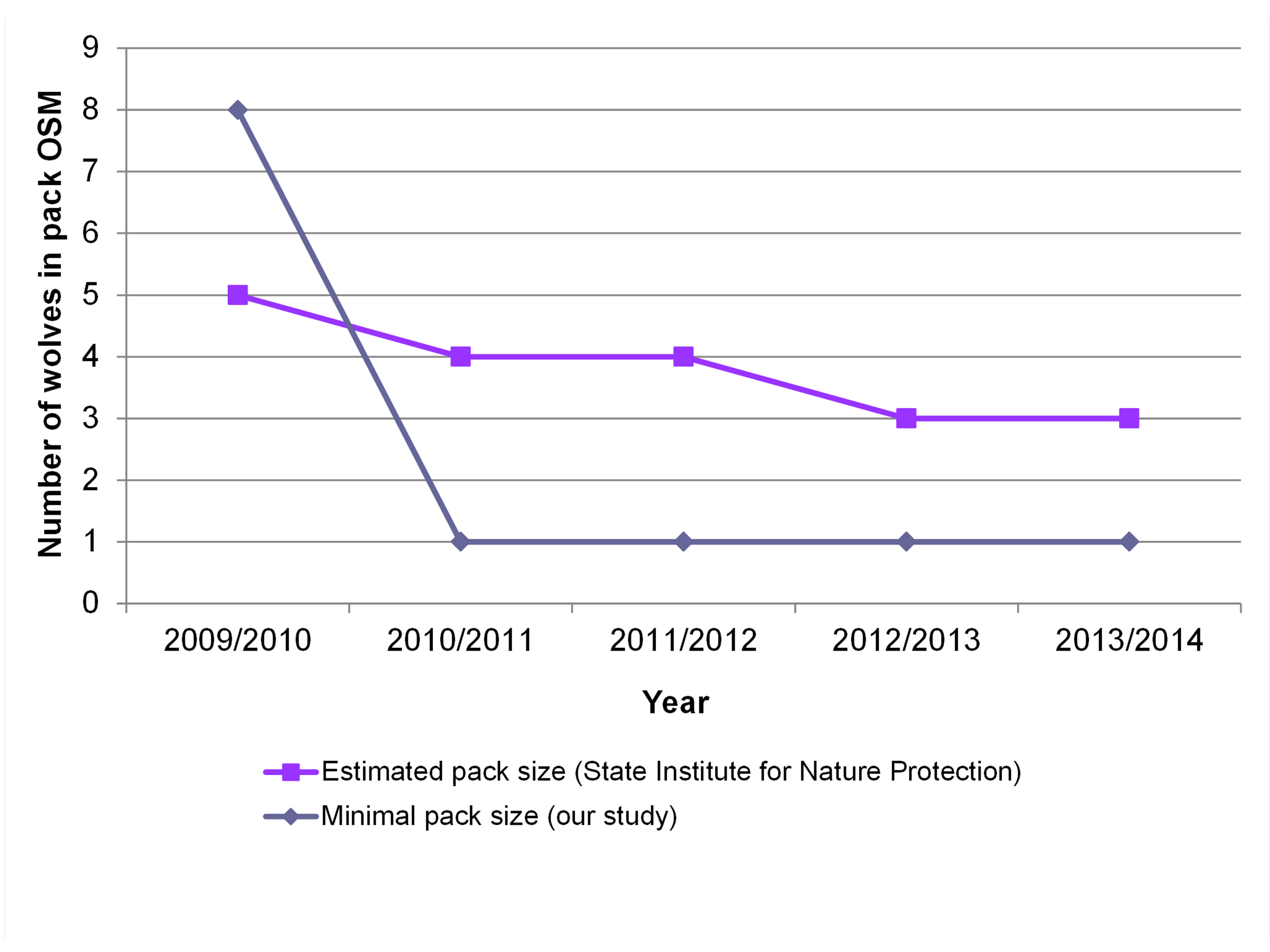

Supplement: S3 Fig — (DOC) [file pone.0156748.s003.doc]
