# Supplementary material for: Camera Traps on Wildlife Crossing Structures as a Tool in Gray Wolf (Canis lupus) Management - Five-Years Monitoring of Wolf Abundance Trends in Croatia
Source: PLoS One. 2016 Jun 21;11(6):e0156748. doi: 10.1371/journal.pone.0156748 (PMC4915698; doi:10.1371/journal.pone.0156748)
Supplement: S1 Table — (DOC) [file pone.0156748.s004.doc]

| **Name** | **Crossing structure type** | **Location** | **Construc-tion year** | **Width / m** | **No. of camera traps** | **Monitoring period** |
| --- | --- | --- | --- | --- | --- | --- |
| **Ivačeno brdo (IB)** | Green bridge | N 45 22.426 E 15 16.161; Gorski kotar | 2003 | 120 | 4 | April 1^st^ 2009 - March 31^st^ 2014 |
| **Medina gora (MG)** |  | N 44 41.916 E 15 23.850; Lika | 2004 | 125 |  |  |
| **Varošina**  **(VAR)** |  | N 44 37.842 E 15 26.383; Lika | 2004 | 125 |  |  |
| **Osmakovac (OSM)** |  | N 43 35.291 E 16 26.512; Dalmatia | 2004 | 200 |  |  |
| **Rošca (RO)** |  | N 43 33.560 E 16 41.643; Dalmatia | 2007 | 150 |  |  |
| **Konšćica (KON)** |  | N 43 31.789 E 16 43.868; Dalmatia | 2007 | 150 |  |  |
| **Graba (GRABA)** | Viaduct | N 44 31.092 E 15 28.512; Lika | 2004 | 64 | 2 | April 1^st^ 2010 - March 31^st^ 2012 |
| **Suvaja (SUV)** |  | N 44 31.012 E 15 28.998; Lika | 2004 | 81 | 3 |  |
| **Mandarića potok (MP)** |  | N 44 30.863 E 15 30.292; Lika | 2004 | 23 | 1 |  |
| **Grabara (GRABARA)** |  | N 44 26.102 E 15 37.527; Lika | 2004 | 114 | 4 |  |
